# Opioidergic and dopaminergic manipulation of gambling tendencies: a preliminary study in male recreational gamblers

**DOI:** 10.3389/fnbeh.2013.00138

**Published:** 2013-10-07

**Authors:** Roseline I. Porchet, Linde Boekhoudt, Bettina Studer, Praveen K. Gandamaneni, Nisha Rani, Somashekar Binnamangala, Ulrich Müller, Luke Clark

**Affiliations:** ^1^Department of Psychology, University of CambridgeCambridge, UK; ^2^Department of Psychology, Behavioural and Clinical Neuroscience Institute, University of CambridgeCambridge, UK; ^3^Institute of Cognitive Neuroscience, University College LondonLondon, UK; ^4^Department of Psychiatry, University of CambridgeCambridge, UK; ^5^Cambridgeshire and Peterborough NHS Foundation TrustCambridge, UK

**Keywords:** naltrexone, haloperidol, pathological gambling, addiction, reward, motivation, decision-making, psychophysiology

## Abstract

Gambling is characterized by cognitive distortions in the processing of chance and skill that are exacerbated in pathological gambling. Opioid and dopamine dysregulation is implicated in pathological gambling, but it is unclear whether these neurotransmitters modulate gambling distortions. The objective of the current study was to assess the effects of the opioid receptor antagonist naltrexone and the dopamine D2 receptor antagonist haloperidol on gambling behavior. Male recreational gamblers (*n* = 62) were assigned to receive single oral doses of naltrexone 50 mg, haloperidol 2 mg or placebo, in a parallel-groups design. At 2.5 h post-dosing, participants completed a slot machine task to elicit monetary wins, “near-misses,” and a manipulation of personal choice, and a roulette game to elicit two biases in sequential processing, the gambler's fallacy and the hot hand belief. Psychophysiological responses (electrodermal activity and heart rate) were taken during the slot machine task, and plasma prolactin increase was assessed. The tasks successfully induced the gambling effects of interest. Some of these effects differed across treatment groups, although the direction of effect was not in line with our predictions. Differences were driven by the naltrexone group, which displayed a greater physiological response to wins, and marginally higher confidence ratings on winning streaks. Prolactin levels increased in the naltrexone group, but did not differ between haloperidol and placebo, implying that naltrexone but not haloperidol may have been functionally active at these doses. Our results support opioid modulation of cognition during gambling-like tasks, but did not support the more specific hypothesis that naltrexone may act to ameliorate cognitive distortions.

## Introduction

Gambling is a widespread form of recreational risk-taking that becomes excessive and pathological in a subset of the population (around 1%; Wardle et al., 2010). Pathological gambling is increasingly viewed as a “behavioral addiction” and has been reclassified within the Addictions category in the DSM-5 (Petry et al., 2013). Recent work on pathological gambling has studied its underlying neurobiological basis, highlighting the similarities with substance use disorders (Potenza, 2008) and focusing on the neuroimaging of reward-based tasks (Limbrick-Oldfield et al., 2013) and changes in neurotransmitter function (Leeman and Potenza, 2012). A distinct cognitive approach to gambling has emphasized the role of erroneous thinking styles (“cognitive distortions”) during gambling (Ladouceur and Walker, 1996; Clark, 2010): gamblers experience a variety of biases and erroneous thoughts during play, pertaining in particular to their perceived level of skill in controlling the outcomes (“the illusion of control”; Langer, 1975) and their tendency to detect patterns in random sequences (“the Gambler's Fallacy”; Oskarsson et al., 2009). While the gambling cognitions are apparent in non-problem gamblers and student populations, the overall level of distorted thinking is elevated in people with gambling problems (Miller and Currie, 2008; Emond and Marmurek, 2010; Michalczuk et al., 2011) and these cognitions can be targeted effectively by cognitive-behavioral therapies (Fortune and Goodie, 2012). The neurobiological mechanisms that underlie these gambling-related distortions have received minimal attention to date, and the aim of the present study was to examine their pharmacological basis, looking at dopamine and opioid receptor manipulations, in a sample of mild recreational gamblers.

The opioid system is the target of growing interest in pathological gambling, primarily on the basis of clinical trials showing significant benefits of the opioid receptor antagonists naltrexone and nalmefene on gambling symptom severity and self-reported craving (Kim et al., 2001; Grant et al., 2006, 2008). These medications are well established in the clinical management of opiate and alcohol dependence (O'Brien, 2005). Preclinical evidence indicates that opioid receptors are distributed widely in the mesolimbic system, and can modulate dopamine transmission (Spanagel et al., 1992). Endogenous opioids are implicated particularly in hedonic aspects of reward processing (Pecina et al., 2006; Barbano and Cador, 2007). Of relevance to gambling behavior, a pharmacological fMRI study of the μ-opioid antagonist naloxone found attenuated reward-related responses in the ventral striatum, and enhanced loss-related activity in the medial prefrontal cortex, on a wheel of fortune task in healthy volunteers (Petrovic et al., 2008). Thus, the treatment effect in pathological gambling may be mediated by a dual action of enhancing aversive processing and attenuating positive processing of gambling outcomes. The present study employed the opioid receptor antagonist naltrexone, which is a competitive antagonist at μ- and κ-opioid receptors, and to a lesser extent at δ-opioid receptors (Kreek, 1996). We used a 50 mg single dose that is widely used in other cognitive studies in healthy volunteers (Katzen-Perez et al., 2001; Mitchell et al., 2007; Boettiger et al., 2009).

Dopamine dysregulation has also been indicated in problem gambling, based on genetic data (Lobo and Kennedy, 2009) and studies measuring peripheral markers (Bergh et al., 1997; Meyer et al., 2004), as well as the provocative syndrome in Parkinson's Disease where medications acting at the dopamine D2/D3-receptor are linked to the emergence of disordered gambling as a side-effect (Voon et al., 2009; Djamshidian et al., 2011). Dynamic PET studies with the dopamine D2/D3 radiotracer [11C]raclopride have confirmed that monetary reinforcement induces dopamine release in healthy volunteers performing gambling-like tasks (Zald et al., 2004; Martin-Soelch et al., 2011), and the magnitude of dopamine release is elevated in at least a subset of patients with pathological gambling (Steeves et al., 2009; Linnet et al., 2011; Joutsa et al., 2012). In addition, acute administration of the dopamine stimulant amphetamine, and the D2-receptor antagonist haloperidol, were both seen to modulate gambling tendencies in pathological gamblers (Zack and Poulos, 2004, 2007). In the present study, we sought to manipulate dopamine transmission with haloperidol, a first generation antipsychotic with high D2 binding affinity in the striatum (Kapur et al., 1996; Xiberas et al., 2001). We selected a low (2 mg) dose of haloperidol that we expected to act preferentially on the presynaptic D2 auto-receptors to *increase* dopamine transmission (Frank and O'Reilly, 2006).

We examined a number of gambling variables that can be elicited with laboratory tasks. We used a slot machine task that delivered unpredictable monetary wins as well as “near-miss” outcomes: non-wins that are spatially proximal to a jackpot win (Reid, 1986). Relative to “full-misses,” near-misses are rated as unpleasant but increase motivations to continue gambling, despite their objective non-win status (see also Kassinove and Schare, 2001). Previous neuroimaging of this task showed that near-misses recruited overlapping brain circuitry to the win outcomes, including the ventral striatum and insula, in both healthy volunteers and regular gamblers (Clark et al., 2009; Chase and Clark, 2010). In the present study, we measured the subjective response to these wins and near-misses with trial-by-trial ratings. We also recorded psychophysiological activity following these outcomes using electrodermal activity (EDA) and heart rate (HR) recording, which have established sensitivity to gambling outcomes (Dixon et al., 2011; Lole et al., 2012; Studer and Clark, 2011; Clark et al., 2012a). In addition, the slot machine task measures one example of illusory control, the effect of personal choice, by comparing the expectancies of winning under conditions where the participant either chose, or was not able to choose, the “play icon.” Subjects rate their expectancy of winning as higher on participant-chosen trials (Clark et al., 2009, 2012a), and fMRI signals to monetary wins are enhanced under this choice manipulation (Coricelli et al., 2005; Studer et al., 2012).

We also administered a second task, based upon roulette, which involved binary predictions of red or black outcomes and a subsequent confidence rating (Ayton and Fischer, 2004). The Gambler's Fallacy is observed as the reduced choice of one color (e.g., red) after a “run” of consecutive outcomes of that color (e.g., four successive reds). In addition, participant's confidence ratings are sensitive to their prediction accuracy, with “streaks” of consecutive correct guesses (i.e., wins) increasing self-reported confidence, and incorrect predictions (i.e., a losing streak) leading to decreased confidence. These are known as “hot hand” effects (Gilovich et al., 1985; Ayton and Fischer, 2004). Past neuroimaging studies found modulation of caudate, insula and medial prefrontal cortex activity by streaks of wins and losses in binary choice games (Elliott et al., 2000; Akitsuki et al., 2003).

As a preliminary investigation, we examined the effects of haloperidol and naltrexone on these gambling variables in a group of healthy male volunteers, who reported recreational gambling involvement. There is evidence that both gambling-related cognitive distortions, and problem gambling symptom severity, exist on a continuum, such that recreational gamblers are considered at some degree of risk for later problematic gambling (Toce-Gerstein et al., 2003; Raylu and Oei, 2004). Rates of gambling involvement and the prevalence of pathological gambling are typically higher in males (Bland et al., 1993; Shaffer et al., 1999).

The overarching hypothesis was that the gambling cognitions under scrutiny would be modulated by the dopamine and opioid-based treatments. Given preclinical evidence that μ-opioid blockade exerts a downstream effect on dopamine transmission (Spanagel et al., 1992), we were further interested in the overlap between the cognitive variables affected by naltrexone and haloperidol. Previous work afforded a number of more specific predictions. First, there are some indications that dopamine may modulate near-miss effects and illusory control, specifically. Using a rodent version of a slot machine, amphetamine and the dopamine D2 agonist quinpirole increased erroneous lever presses on a game with near-misses (2 of 3 identical symbols) (Winstanley et al., 2011). Dopamine is also implicated in perceptions of control (Declerck et al., 2006; Redgrave and Gurney, 2006); for example, levodopa increased the sense of agency (“action-effect binding”) on a timing task in patients with Parkinson's Disease (Moore et al., 2010). As such, we predicted that the low dose of haloperidol would potentiate subjective and physiological responses to win and near-miss outcomes, and enhance the influence of personal choice, on the slot machine task. Second, drawing on Petrovic et al. (2008), we predicted that the naltrexone group would show attenuated responses to winning outcomes, coupled with enhanced negative processing (affect following near-misses) on the two tasks. Given the lack of past work to guide predictions about neurotransmitter effects on the Roulette task, these data were analyzed in an exploratory manner.

## Methods

### Participants

Male participants (*n* = 62) were recruited through the University and community advertisements. Participants were aged 18–49 years, and reported past year gambling involvement and at least 5 lifetime gambling experiences. Exclusion criteria (confirmed through telephone interview): a score = 8 (indicative of probable pathological gambling) on the Problem Gambling Severity Index (Ferris and Wynne, 2001), significant neurological or physical illness, current or past mental health problems, including substance use and heavy smoking (>10 cigarettes/day). The study was approved by the Cambridgeshire 4 Research Ethics Committee (10/H0305/79). All participants gave written informed consent and were paid £35 for their participation (plus a task-related bonus of £6).

### Study design

The study was a double-blind, parallel-groups, placebo-controlled design, involving a single session at a clinical research facility. Subjects were randomly allocated to the three treatment groups: 2 mg haloperidol, 50 mg naltrexone or placebo (microcritalline cellulose) hidden in identical gelatine capsules. Upon arrival, a urine sample was taken to confirm absence of recent opiate use, and participants completed trait questionnaires assessing impulsivity (UPPS-P; Cyders et al., 2007) and susceptibility to gambling biases (Gambling Related Cognition Scale; Raylu and Oei, 2004). Participants also completed the electronic Mini International Neuropsychiatric Interview (eMINI) (Sheehan et al., 1998) for further investigation of current and lifetime psychiatric disorders. Mental health problems were detected in 17 participants; 7 subjects in the placebo group (alcohol dependence *n* = 4, obsessive-compulsive disorder *n* = 1, hypomanic episode *n* = 1, bulimia nervosa *n* = 1), 7 subjects in the haloperidol group (alcohol dependence *n* = 1, alcohol abuse *n* = 2, obsessive-compulsive disorder *n* = 2, cocaine abuse *n* = 1, generalized anxiety disorder *n* = 1); and 3 subjects in the naltrexone group (alcohol dependence *n* = 1, hypomanic episode *n* = 1, major depressive episode *n* = 1). The proportion of participants meeting eMINI diagnoses did not differ across the three treatment groups (χ^2^ = 2.77, *p* = 0.25). Given that participants had disclosed no past or current mental health problems in the telephone interviews, we cannot rule out the possibility that the eMINI detections were false positives.

Following dosing, participants rested for 2.5 h to allow drug absorption. This timing was based upon pharmacokinetic data showing that haloperidol reaches maximal plasma concentrations after 3 h (plasma half-life: 24 h) (Darby et al., 1995), whereas naltrexone reaches maximal plasma concentration after 45 min with a plasma half-life of 4 h (Crabtree, 1984; Meyer et al., 1984). After this rest period, volunteers completed the Slot Machine Task (Clark et al., 2009) with concurrent psychophysiological monitoring of HR and EDA, followed by the Roulette Task (Ayton and Fischer, 2004). Blood samples were taken pre-dosing (T1) and at the start of the testing period (T2, +2.5 h) to measure serum prolactin levels as a marker of dopaminergic tone (Ben-Jonathan and Hnasko, 2001). Blood pressure (BP) and HR were measured with a wrist cuff, and mood was measured with Visual Analogue Scales (Bond and Lader, 1974), at T1, T2, and on completion of testing (T3, +4 h). VAS data were unavailable for a single subject.

### Prolactin analysis

Blood samples (4.7 ml) were centrifuged at 4000 rpm for 5 min at room temperature to obtain serum and then distributed into two aliquots of about 1.5 ml. The samples were frozen at −80°C until analysis. Prolactin levels were analyzed by the National Institute for Health Research Cambridge Biomedical Research Center Core Biochemistry Assay Laboratory, Addenbrooke's hospital, and were tested with immunofluorometric assay (ADVIA Centaur prolactin assay, Siemens). Results are reported in mU/L. Prolactin samples were unavailable or contaminated by macroprolactin in two subjects.

### Tasks

#### Slot machine task

**Figure 1:**
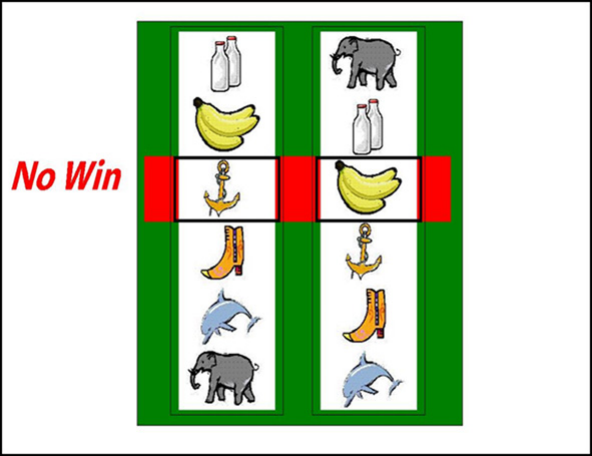
**The slot machine task displayed two reels, with the same six icons on each reel**. Each trial involved a fixed £0.15 p wager. After a selection phase in which either the computer or the participant chose one of the icons on the left reel as the “play icon,” the right reel spun for a variable anticipation phase. The right reel decelerated and came to a standstill. If the right reel stopped on the chosen play icon, i.e., the reels were aligned on the central payline, the subject won £1. If the right reel stopped on a different icon (5/6 trials), the participant lost their wager. In the analysis of these non-wins, we distinguished near-misses (with the play icon either side of the payline) and full-misses (with the play icon more than one position from the payline).

Participants completed 60 trials (following 4 practice trials) on a simplified two-reel slot machine task, described in detail in Clark et al. (2009) (see Figure 1). Psychophysiological signals (EDA and HR) were monitored during the task using a Biopac MP36 (see below). The screen background color (white or black) designated two choice conditions: either participant-chosen trials, in which the participant selected the “play icon” on the left reel by scrolling the reel up or down, and computer-chosen trials, in which the play icon was selected automatically. Following icon selection, the right reel spun and decelerated (mean spin time: 4.2 s) to deliver a win (£1), near-miss, or full-miss outcome (outcome duration 6 s). Current earnings were displayed in the inter-trial interval (duration 5 s), with an initial endowment of £5. The outcomes and choice condition (participant-chosen, computer-chosen) occurred in a fixed pseudo-random sequence such that wins occurred on 1/6, and near-misses on 1/3 trials. As a consequence of the fixed sequence, all participants completed the task with £6, which they received as a bonus.

On each trial, three Likert ratings were taken: following icon selection, “How do you rate your chances of winning?” (0 to +100), and following the outcome, “How pleased are you with the result?” (−100 to +100) and “How much do you want to continue to play?” (0 to +100).

#### Roulette task

**Figure 2:**
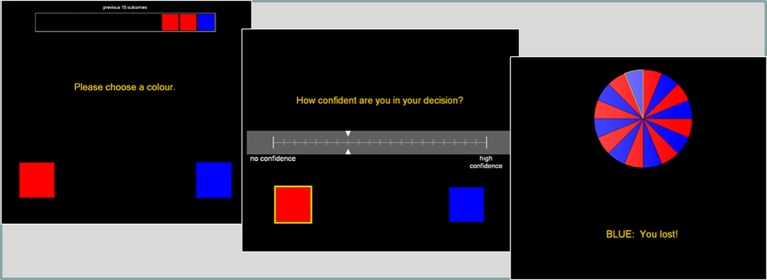
**The roulette task presented a color choice (red or blue) on each trial, followed by a confidence rating**. The roulette wheel then spun, and the outcome and feedback were presented. A history bar across the top of the screen indicated the last ten outcomes, to alleviate any working memory requirements.

This binary choice task was modified from Ayton and Fischer (2004). The roulette wheel displayed an equal number of red and blue segments (see Figure 2), and on each trial, the participant first guessed red or blue, and then gave a confidence rating on 21-point scale. A history bar during the color choice presented the 10 previous outcomes, to minimize working memory demands that may be independently affected by the drug treatments.

Following the color choice and confidence rating, the wheel spun for 800–1200 ms, and the outcome was presented (e.g., “Blue: you win”). Participants received £0.10 for correct guesses, with no reinforcement (i.e., losses) for incorrect guesses. Participants completed 3 practice trials, followed by a total of 90 trials, using a pre-specified color sequence in order to deliver runs of 1–5 consecutive outcomes of the same color. This fixed sequence had an equal probability of either color, and a probability of alternation of 0.48 (see Oskarsson et al., 2009 for derivation). We refer to consecutive outcomes of the same color as “outcome runs” (i.e., blue, red, red, red is an outcome run of length 3), and consecutive correct or incorrect predictions as “feedback streaks.” Two dependent variables were derived: (1) the probability of choosing either color as a function of the outcome run of that color, indicative of the Gambler's Fallacy, (2) the confidence rating as a function of feedback streak, indicative of the Hot Hand Beliefs.

### Psychophysiological measurement

During the slot machine task, electrodermal activity (EDA) and HR calculated from electrocardiogram (ECG) were recorded via a BIOPAC MP36 unit (BIOPAC Systems Ltd, Goleta, CA, USA), following methods previously (Clark et al., 2012a,b). The BIOPAC unit, sampling at 1000 Hz, was connected to the stimulus delivery computer and to a second recording computer running AcqKnowledge 4.1 software. Task events were marked on the psychophysiological trace via a parallel port connection. EDA was recorded through fingertip electrodes attached to the index and middle fingers of the non-dominant hand. Heart rate was recorded using ECG electrode patches applied to the right wrist and left ankle. The psychophysiological data were extracted using in-house scripts developed in Microsoft Visual Basic (v6.0): activity on the slot machine task was modeled to the time of outcome delivery, using change from baseline scores calculated from the mean activity in the final 2 s of reel spin. Mean EDA was extracted in 6 × 2 s bins from the onset of the outcome phase. An EDA summary measure was calculated from the maximum change from baseline value in bins 2–4 (i.e., 2–8 s post-outcome), given the typical time-course for EDA changes (Dawson et al., 2000). HR responses were calculated using the median HR in 12 × 0.5 s bins from the onset of the outcome phase. Two HR summary measures isolated the initial HR deceleration component (the minimum value in bins 1–6, i.e., 0–3 s post-outcome, minus the baseline) and the subsequent HR acceleration component (the maximum in bins 7–12, i.e., 3–6 s post-outcome, minus the deceleration minima) (Hodes et al., 1985; Bradley, 2000).

### Statistical analysis

Statistical analysis was performed in SPSS version 19.0. Demographic and trait variables were compared across groups using One-Way ANOVA. Fisher's least significant difference test was used for *post-hoc* comparisons, as is appropriate for 3-group designs (Cardinal and Aitken, 2006). Mood scales, cardiovascular measures, and prolactin levels were assessed with mixed-model ANOVAs including Timepoint as a within-subjects factor.

On the slot machine task, the subjective ratings and psychophysiology summary measures were analyzed with mixed-model ANOVA, with Outcome (wins, near-misses, full-misses) and Choice (participant-chosen, computer-chosen) as within-subjects factors, and Treatment (3 levels: haloperidol, naltrexone, placebo) as a between-subjects factors. Data from the roulette task were analyzed using two mixed-model ANOVAs, with Treatment (3 levels: placebo, haloperidol, naltrexone) as the between-subjects factor. For analysis of color predictions, Outcome Run length was the within-subjects factor. For the analysis of confidence ratings, Feedback Streak length and Outcome (winning, losing) were within-subjects factors. Simple main effects analysis of the roulette task data compared shorter runs/streaks (1–2 successive events) against longer runs/streaks (4–5 successive events). As the feedback streaks were not pre-specified, some subjects did not experience any longer streaks. For participants missing only streaks of length five, we imputed their streak length 4 value for their missing value (this is a conservative approach that underestimates any effect of the longer streaks). Three participants were excluded who did not experience streaks longer than three events. In addition, one further participant was excluded who did not vary either his color choice or confidence ratings across the task.

As the primary aim of this study was to compare the effects of haloperidol and naltrexone relative to the placebo condition, rather than the direct comparison of the two active treatments, the omnibus 3-group model was decomposed using two planned comparisons of the haloperidol group vs. placebo, and the naltrexone group vs. placebo. For all analyses, the Greenhouse-Geisser correction was applied when sphericity assumptions were violated, and the Huynh-Feldt correction was reported when the Greenhouse-Geisser estimate was greater than 0.75 (Cardinal and Aitken, 2006). All tests were thresholded at *p* < 0.05 two-tailed.

## Results

The three treatment groups did not differ significantly in age, years of education, trait gambling distortions, or impulsivity (see Table 1). The overall level of problem gambling was low on the PGSI (mean 1.5; *SD* 1.73; range 0–7; a score = 8 is indicative of probable pathological gambling), but GRCS scores were in range of previous data in recreational gamblers (Raylu and Oei, 2004; Billieux et al., 2012).

**Table 1:** **Participant characteristics and demographic details**.

	**Placebo**	**Haloperidol**	**Naltrexone**	**Test statistic**
	**(*N* = 20)**	**(*N* = 21)**	**(*N* = 21)**	
Age (years)	27.2 (8.0)	26.6 (7.1)	27.1 (8.3)	*F*_(2, 61)_ = 0.04, NS
Education	17.4 (3.1)	16.3 (3.1)	16.7 (2.3)	*F*_(2, 61)_ = 0.68, NS
PGSI	1.6 (1.7)	1.6 (1.8)	1.3 (1.8)	*F*_(2, 61)_ = 0.17, NS
GRCS	47.7 (21.2)	53.4 (15.1)	58.0 (18.1)	*F*_(2, 61)_ = 1.64, NS
UPPS-P	137.8 (17.8)	128.3 (23.4)	129.2 (14.6)	*F*_(2, 61)_ = 1.57, NS

The values are reported in means and standard deviations; PGSI, problem gambling severity index (range 0–27); GRCS, gambling-related cognitions scale (range 23–161), UPPS-P Impulsivity Scale (range 59–236). NS, not significant.

### Prolactin levels

**Figure 3:**
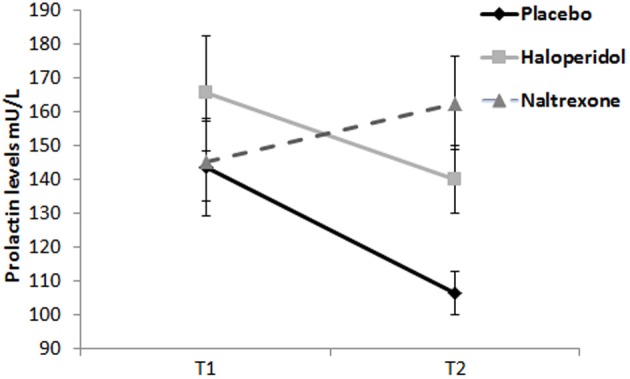
**Prolactin levels (mU/L) at T1 (baseline) and T2 (start of testing period)**. Naltrexone significantly increased prolactin levels. Error bars indicate standard errors of the means.

For plasma prolactin levels, there was a significant Treatment × Time interaction [*F*_(2, 57)_ = 4.09, *p* = 0.022, η^2^_*p*_ = 0.13]. The main effects of Treatment [*F*_(2, 57)_ = 2.42, *p* = 0.098, η^2^_*p*_ = 0.08] and Time [*F*_(1, 57)_ = 3.44, *p* = 0.069, η^2^_*p*_ = 0.06] approached significance. Analysis of change scores (T2 minus T1) indicated prolactin increase in the naltrexone group compared to placebo [*t*_(38)_ = −2.78, *p* = 0.008], consistent with downstream dopaminergic blockade by naltrexone. The haloperidol group did not differ significantly from placebo (*p* > 0.1) (see Figure 3).

### Mood and cardiovascular measures

On the subjective mood ratings, there were no differences between treatment groups (i.e., the Treatment × Time interaction term) for Alertness [*F*_(4, 116)_ = 1.06, NS], Happiness [*F*_(4, 116)_ = 1.70, NS] or Calmness [*F*_(4, 116)_ = 0.09, NS]. Main effects of Time were observed on Alertness [*F*_(2, 116)_ = 23.10, *p* < 0.001, η^2^_*p*_ = 0.29] and Happiness [*F*_(2, 116)_ = 7.26, *p* = 0.001, η^2^_*p*_ = 0.11], reflecting a general decrease over time across all groups.

On the cardiovascular measures, there were no differences between treatment groups (i.e., Treatment × Time interactions) on HR [*F*_(4, 116)_ = 1.07, NS], systolic BP [*F*_(3.3, 96.2)_ = 1.76, NS] or diastolic BP [*F*_(4, 116)_ = 1.65, NS]. Systolic BP and HR decreased over time across all groups [main effect of Time: *F*_(1.7, 96.2)_ = 3.92, *p* < 0.030, η^2^_*p*_ = 0.06; *F*_(2, 116)_ = 45.49, *p* < 0.001, η^2^_*p*_ = 0.44, respectively].

### Slot machine task

#### Subjective effects of wins and near-misses

On the ratings of “pleased with outcome,” the omnibus ANOVA revealed a significant main effect of Outcome [*F*_(1.0, 59.0)_ = 189.66, *p* < 0.001, η^2^_*p*_ = 0.77], such that participants were more pleased after wins compared to near-misses [*t*_(59)_ = 13.15, *p* < 0.001] and full-misses [*t*_(59)_ = 13.40, *p* < 0.001] (see Table 2). Near misses were more pleasant than full misses [*t*_(59)_ = 2.19, *p* = 0.033]. An Outcome × Choice interaction was observed [*F*_(1.5, 84.0)_ = 4.32, *p* = 0.026, η^2^_*p*_ = 0.07], such that for near-misses and full-misses, participant-chosen outcomes were significantly less pleasant than computer-selected outcomes [*t*_(59)_ = 2.32, *p* = 0.024; *t*_(59)_ = 2.84, *p* = 0.006; respectively], whereas for wins, pleasantness ratings did not differ by choice condition [*t*_(59)_ = 1.46, *p* = 0.149]. An Outcome × Treatment interaction was observed [*F*_(2.1, 59.0)_ = 4.15, *p* = 0.020, η^2^_*p*_ = 0.13], driven by an effect of haloperidol [haloperidol model: *F*_(1.0, 39.1)_ = 6.56, *p* = 0.014, η^2^_*p*_ = 0.15; naltrexone model: *F*_(1.0, 38.6)_ = 0.85, NS]. The haloperidol group rated higher pleasure after wins [*t*_(38)_ = −2.20, *p* = 0.034] and greater unpleasantness after non-wins [near-misses: *t*_(38)_ = 2.16, *p* = 0.038; full-misses: *t*_35.7_ = 2.36, *p* = 0.024] compared to the placebo group (see Table 2). Thus, on a subjective rating, haloperidol appeared to potentiate both the positive affect to winning as well as negative affect following non-winning outcomes.

**Table 2:** **Subjective ratings on the slot machine task**.

	**Placebo**	**Haloperidol**	**Naltrexone**
**“CHANCES OF WINNING?”**			
Participant	38.8 (19.2)	46.0 (17.9)	40.9 (16.8)
Computer	32.7 (16.7)	36.7 (15.2)	36.5 (15.2)
**“PLEASED WITH RESULT?”**			
Win, participant	32.0 (31.3)	56.6 (30.7)	49.9 (27.1)
Win, computer	34.1 (26.3)	51.2 (35.5)	43.4 (25.8)
Near-miss, participant	−28.8 (21.2)	−49.9 (27.7)	−28.4 (19.7)
Near-miss, computer	−29.2 (23.9)	−42.9 (28.4)	−24.9 (17.6)
Full-miss, participant	−27.8 (20.1)	−47.2 (28.7)	−26.1 (18.9)
Full-miss, computer	−26.4 (19.3)	−42.6 (27.9)	−21.8 (16.6)
**“CONTINUE TO PLAY?”**			
Win, participant	51.8 (24.6)	60.1 (23.1)	62.3 (13.5)
Win, computer	52.2 (24.4)	62.5 (20.6)	59.2 (12.7)
Near-miss, participant	45.2 (21.3)	56.3 (19.8)	53.6 (12.1)
Near-miss, computer	40.5 (24.7)	51.4 (21.3)	52.2 (11.1)
Full-miss, participant	42.2 (23.1)	51.1 (22.9)	52.3 (12.0)
Full-miss, computer	44.3 (21.9)	53.3 (21.4)	53.5 (11.4)

Values are reported as mean (SD), separated by the participant-chosen condition and the computer-chosen condition.

**Figure 4:**
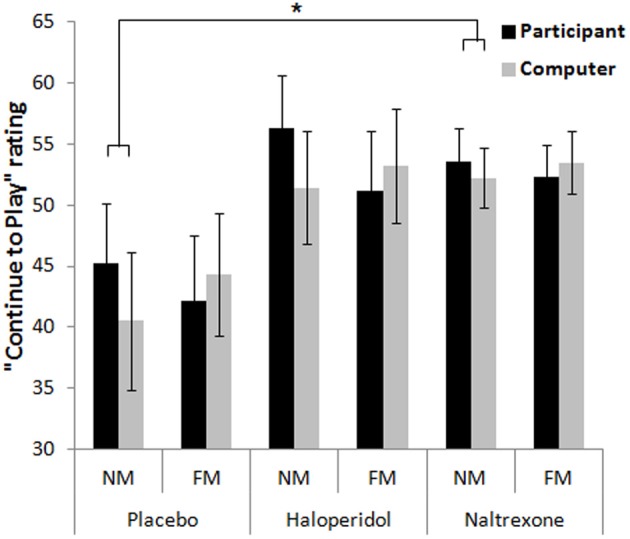
**Motivational ratings on the slot machine task showed an outcome (near-miss, full-miss) by control (participant-chosen, computer-chosen) interaction, whereby participant-chosen near-misses increased motivation to continue relative to the computer-chosen near-misses, in the placebo group (and haloperidol group), and this was attenuated in the naltrexone group**. Error bars indicate standard error of the mean. ^*^p < 0.08.

On the rating of “continue to play,” the omnibus ANOVA revealed a significant main effect of Outcome [*F*_(1.2, 67.0)_ = 45.5, *p* < 0.001, η^2^_*p*_ = 0.44], reflecting higher ratings after wins compared to non-wins [near-misses: *t*_(59)_ = 6.60, *p* < 0.001; full-misses: *t*_(59)_ = 7.53, *p* < 0.001]. There was an Outcome × Choice interaction [*F*_(2, 114)_ = 13.5, *p* < 0.001, η^2^_*p*_ = 0.19]: the desire to play was higher after participant-chosen near-misses, compared to computer-chosen near-misses [*t*_(59)_ = 5.00, *p* < 0.001], and participant-chosen full-misses [*t*_(59)_ = 4.78, *p* < 0.001], as previously observed on this task (Clark et al., 2009, 2012a,b). There was also an Outcome × Choice × Treatment interaction [*F*_(4, 114)_ = 3.39, *p* = 0.012, η^2^_*p*_ = 0.11], driven by an effect of naltrexone [*F*_(2, 74)_ = 3.57, *p* = 0.033, η^2^_*p*_ = 0.09] [haloperidol model: *F*_(2, 76)_ = 0.42, NS]. In the placebo group, the participant-chosen near-misses were rated as more motivating than either computer-chosen near-misses [*t*_(18)_ = 3.27, *p* < 0.001] or participant-chosen full-misses [*t*_(18)_ = 3.66, *p* < 0.001]. These differences were not observed in the naltrexone group (all *p*s > 0.1) and the calculated difference score between participant-chosen and computer-chosen near-misses was marginally higher in the placebo group than in the naltrexone group [*t*_(37)_ = 1.80, *p* = 0.08]. Thus, naltrexone had a modest effect of attenuating the motivational ratings after self-selected near-misses (see Figure 4).

#### Psychophysiological responses to wins and near-misses

**Figure 5:**
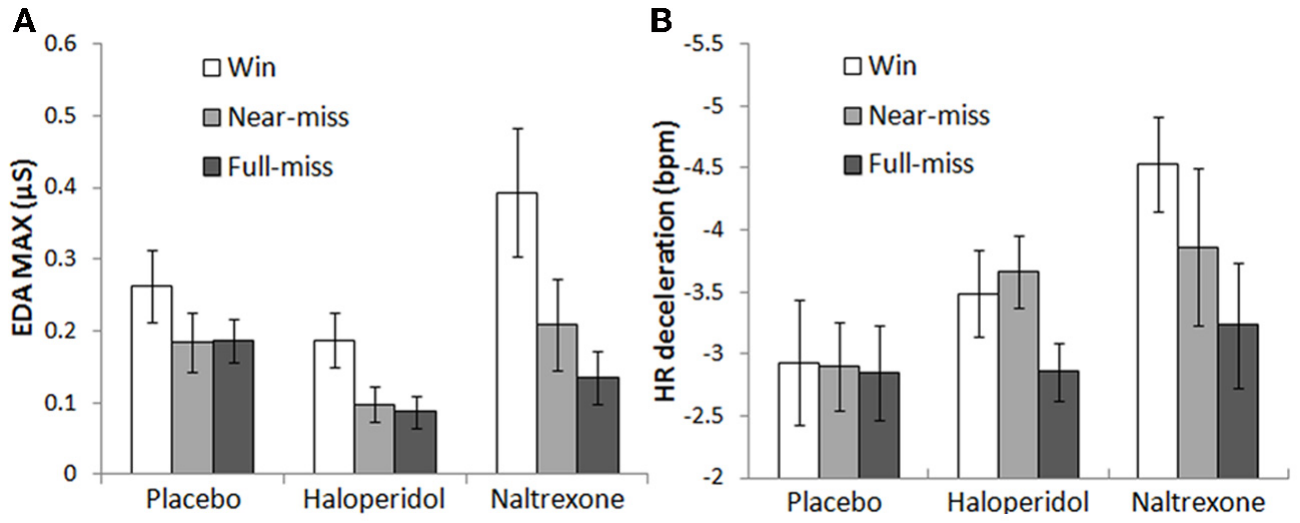
**Psychophysiological responses on the slot machine task: (A) Naltrexone significantly increased post-outcome EDA following wins compared to both non-win outcomes (collapsing across choice conditions)**. **(B)** The naltrexone group displayed marginally elevated post-outcome HR deceleration following wins compared to non-wins (collapsing across choice conditions). Error bars indicate standard error of the mean.

For EDA max, there were significant main effects of Outcome [*F*_(1.4, 77.4)_ = 24.8, *p* < 0.001, η^2^_*p*_ = 0.31] and Choice [*F*_(1, 56)_ = 28.0, *p* < 0.001, η^2^_*p*_ = 0.33]. Across all groups, participants experienced higher EDA responses after wins compared to non-wins [near-misses: *t*_(58)_ = 5.42, *p* < 0.001; full-misses: *t*_(58)_ = 5.14, *p* < 0.001]. There was a marginal increase in EDA after near-misses in comparison to full-misses [*t*_(58)_ = 1.98, *p* = 0.053]. Participants showed higher EDA on participant-chosen outcomes compared to computer-chosen outcomes [wins: *t*_(58)_ = 2.06, *p* = 0.044; near-misses: *t*_(58)_ = 4.32, *p* < 0.001; full-misses: *t*_(58)_ = 2.72, *p* = 0.009]. There was a significant Outcome × Treatment interaction [*F*_(2.8, 77.4)_ = 3.52, *p* = 0.022, η^2^_*p*_ = 0.11], which was driven by the naltrexone group [*F*_(1.4, 51.9)_ = 5.09, *p* = 0.018, η^2^_*p*_ = 0.12] [haloperidol model: *F*_(1.3, 46.0)_ = 0.15, NS]. The EDA change to wins relative to full-misses was greater in the naltrexone group than the placebo group [*t*_(37)_ = −2.47, *p* = 0.018], as well as marginally higher for the near-miss vs. full-miss change score [*t*_(37)_ = −1.80, *p* = 0.081] (see Figure 5A). Thus, naltrexone increased the physiological responsiveness to wins in comparison to full-misses (Table 3).

**Table 3:** **Psychophysiological responses to outcomes on the slot machine task (change scores from baseline)**.

	**Placebo**	**Haloperidol**	**Naltrexone**
**EDA MAX**			
Win, participant	0.30 (0.23)	0.20 (0.17)	0.42 (0.42)
Win, computer	0.23 (0.22)	0.18 (0.21)	0.36 (0.44)
Near-miss, participant	0.22 (0.19)	0.13 (0.14)	0.25 (0.30)
Near-miss, computer	0.15 (0.18)	0.07 (0.12)	0.17 (0.30)
Full-miss, participant	0.21 (0.17)	0.10 (0.11)	0.16 (0.21)
Full-miss, computer	0.16 (0.13)	0.7 (0.10)	0.11 (0.15)
**HR DECELERATION**			
Win, participant	−2.4 (1.8)	−3.6 (2.2)	−4.7 (3.0)
Win, computer	−3.4 (2.9)	−3.4 (2.0)	−4.4 (4.7)
Near-miss, participant	−3.0 (1.9)	−3.6 (1.6)	−3.9 (2.6)
Near-miss, computer	−2.8 (2.0)	−3.7 (1.4)	−3.8 (3.6)
Full-miss, participant	−2.7 (1.4)	−2.9 (1.0)	−3.2 (2.1)
Full-miss, computer	−3.0 (2.0)	−2.8 (1.3)	−3.3 (2.5)
**HR ACCELERATION**			
Win, participant	5.8 (3.6)	6.1 (3.2)	7.3 (4.8)
Win, computer	5.0 (2.9)	5.6 (3.4)	6.2 (5.2)
Near-miss, participant	6.1 (2.7)	6.5 (2.9)	7.1 (5.1)
Near-miss, computer	5.8 (2.3)	6.9 (3.5)	7.5 (5.4)
Full-miss, participant	5.7 (2.4)	6.1 (3.3)	6.4 (5.0)
Full-miss, computer	4.9 (1.8)	5.3 (2.5)	5.7 (4.1)

Values are reported as mean (SD), separated by participant-chosen condition and the computer-chosen condition. EDA, electrodermal activity, in μS, where the Max refers to the maximum value across bin 2–4 (i.e., 2–8 s post-outcome) minus the pre-trial baseline. HR, heart rate, in beats per minute, where the Deceleration value refers to the minimum value in bins 1–6 (i.e., 0–3 s post-outcome) minus the baseline; and the subsequent HR acceleration component refers to the maximum in bins 7–12 (i.e., 3–6 s post-outcome) minus the deceleration minima.

On HR deceleration, there was a main effect of Outcome [*F*_(2, 106)_ = 6.44, *p* = 0.002, η^2^_*p*_ = 0.11], with greater HR decelerations after wins and near-misses in comparison to full-misses [*t*_(55)_ = −3.23, *p* = 0.002; *t*_(55)_ = −3.03, *p* = 0.004; respectively]. A trend Outcome × Treatment interaction was observed [*F*_(4, 106)_ = 2.11, *p* = 0.085, η^2^_*p*_ = 0.074], driven by an effect of naltrexone [*F*_(2, 70)_ = 2.86, *p* = 0.064, η^2^_*p*_ = 0.08] [haloperidol model: *F*_(2, 68)_ = 1.64, NS]. The HR deceleration to wins relative to full-misses was greater in the naltrexone group than the placebo group [*t*_(35)_ = 2.17, *p* = 0.03] (see Figure 5B), similar to the EDA effect. For HR acceleration, there was a significant effect of Outcome [*F*_(1.8, 93.4)_ = 8.12, *p* = 0.001, η^2^_*p*_ = 0.13], reflecting higher HR acceleration after near-misses relative to wins [*t*_(55)_ = 2.45, *p* = 0.018] and full-misses [*t*_(55)_ = 4.99, *p* < 0.001]. There was also a main effect of Choice [*F*_(1, 53)_ = 4.17, *p* = 0.046, η^2^_*p*_ = 0.07], indicating higher HR acceleration for participant-chosen outcomes compared to computer-chosen outcomes [wins: *t*_(55)_ = 2.02, *p* = 0.048; full-misses: *t*_(55)_ = 2.89, *p* = 0.006]. The HR acceleration effects did not interact with Treatment group.

#### Subjective effects of personal choice (the illusion of control)

On the ratings of “chances of winning,” participants reported a greater expectancy of winning when they chose the play icon, compared to the computer-chosen condition [*F*_(1, 57)_ = 44.59, *p* < 0.001, η^2^_*p*_ = 0.44]. This effect did not vary across treatment groups [Treatment × Choice: *F*_(2, 57)_ = 2.19, NS; Treatment: *F*_(2, 57)_ = 0.59, NS] (see Table 2).

### Roulette task

#### Gambler's fallacy

The analysis of color choice yielded a main effect of Run Length [*F*_(1, 55)_ = 7.84, *p* = 0.007, η^2^_*p*_ = 0.13], reflecting decreased choice of choosing either color after a longer run of that color (*M* = 43.1, *SD* = 23.6) compared to a short run (*M* = 50.9, *SD* = 9.5). This represents a typical Gambler's fallacy pattern. Treatment group did not moderate this effect [Run Length × Treatment: *F*_(2, 55)_ = 1.07, NS; Treatment: *F*_(2, 55)_ = 0.74, NS].

#### Hot hand belief

**Figure 6:**
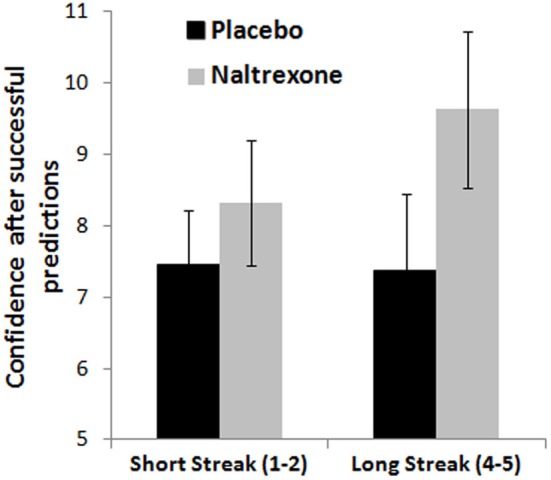
**Confidence ratings on the roulette task as a function of winning streak length show a marginal difference between the naltrexone and placebo groups**. Error bars indicate standard error of the mean.

Analysis of confidence ratings as a function of feedback streak showed a weak effect of Outcome [*F*_(1, 55)_ = 3.34, *p* = 0.073, η^2^_*p*_ = 0.06], whereby confidence was higher after correct predictions compared to incorrect predictions, in line with the hot hand belief. The Streak Length × Outcome × Treatment interaction approached significance [*F*_(2, 55)_ = 2.51, *p* = 0.091, η^2^_*p*_ = 0.08]. This effect was driven by the naltrexone group, in which a significant 3-way interaction [*F*_(1, 35)_ = 5.41, *p* = 0.026, η^2^_*p*_ = 0.13] and a trend Outcome × Treatment interaction [*F*_(1, 35)_ = 3.81, *p* = 0.059, η^2^_*p*_ = 0.10] were observed [haloperidol model: *F*_(1, 37)_ = 2.37, NS]. Analysing winning and losing streaks separately in the naltrexone model, the Streak Length × Treatment interaction approached significance for wins [*F*_(1, 35)_ = 3.43, *p* = 0.073, η^2^_*p*_ = 0.09], but not for losses [*F*_(1, 35)_ = 1.91, NS], such that the naltrexone group showed a greater increase in confidence on longer winning streaks, compared to placebo (see Figure 6).

## Discussion

In this study, we assessed the effects of the opioid antagonist naltrexone and the dopamine D2-receptor antagonist haloperidol on two gambling tasks in male recreational gamblers. A slot machine task was used to deliver near-miss outcomes, elicit perceptions of control, and to measure physiological responses to winning outcomes. A roulette task was used to study the impact of outcome runs and feedback streaks on choice behavior and confidence ratings, respectively. Collapsing across the three treatment groups, both tasks were reasonably successful at inducing these gambling phenomena. On the slot machine task, the jackpot wins were rated as pleasurable and increased the motivation to play, and the winning outcomes were also associated with increased EDA and HR deceleration, relative to the non-wins. Comparing near-misses to full-misses, we confirmed our previous results on this task, that motivation ratings were higher after near-misses, and this effect depended on personal choice over the gamble (Clark et al., 2009, 2012a). The perceived chances of winning were also higher on participant-chosen trials than computer-chosen trials, consistent with an illusion of control. Near-misses were associated with increased EDA and rebound HR acceleration, as we have described previously (Clark et al., 2012a, 2013). On the roulette task, there was an expected Gambler's Fallacy effect, such that the choice of either color decreased after long runs of that color (i.e., negative recency) (replicating Ayton and Fischer, 2004). There was also a weaker effect of increased confidence after wins compared to losses, consistent with the “Hot Hand” belief (Ayton and Fischer, 2004).

In terms of the pharmacological effects, several differences were observed between the treatment groups, although generally, these were not in line with our predictions. The three groups were demographically matched and did not differ significantly on impulsivity, a relevant personality trait, or level of gambling involvement (PGSI) or trait gambling cognitions (GRCS). Prolactin levels increased in the naltrexone-treated group, but did not differ significantly between the haloperidol group and the placebo group. This implies that the single low dose of naltrexone (50 mg) was functionally active, but that the 2 mg haloperidol dose may not have been. Indeed, on the two gambling tasks, the majority of the detected group differences were between the naltrexone and placebo groups: the naltrexone group had a greater physiological response to winning outcomes on the slot machine task, in terms of EDA (significant) and HR deceleration (marginally significant). On the roulette task, the naltrexone group showed marginally higher confidence ratings after winning streaks compared to the placebo group, indicating a possible enhancement of the hot hand effect. At the same time, the motivational effect of the near-misses on participant-chosen trials was significantly attenuated in the naltrexone group. By contrast, in the haloperidol group, the only observed effect was a greater disparity in pleasantness ratings between the win and non-win outcomes (i.e., a treatment by outcome interaction). Neither group showed differences in the effect of personal control on the slot machine task, or the Gambler's Fallacy on the roulette task.

### Effects of naltrexone on gambling behavior

Based upon the reported clinical efficacy of naltrexone in the treatment of pathological gambling (Kim et al., 2001; Grant et al., 2006, 2008), our overarching hypothesis for the naltrexone group was that cognitive effects characteristic of excessive gambling would be ameliorated by naltrexone. In addition, we predicted that these participants would show blunted responses to wins (c.f. Petrovic et al., 2008). Our data indicated that naltrexone did modulate the responsivity to wins, but in the opposite direction to that predicted: the naltrexone group displayed *higher* EDA following wins, and this hyper-reactivity was substantiated by a trend effect for HR deceleration. *Prima facie*, these results are difficult to reconcile with the substantial literature reporting that opioid blockade reduces reward processing in laboratory models (Drewnowski et al., 1995; Zhu et al., 2011; Langleben et al., 2012a), and reduces cravings and drug self-administration in groups with substance use disorders (Davidson et al., 1999; Drobes et al., 2004; Myrick et al., 2008; Langleben et al., 2012b; Miranda et al., 2013).

A number of methodological differences may be pertinent here, and may be useful to inform the design of future experiments. A key point is that our participants were recreational gamblers with modest levels of gambling involvement; it is possible that pathological gamblers may show a qualitatively different response to opioid blockade. Our decision to use recreational gamblers was based on several factors: the ease of recruitment to achieve sufficient group sizes, ethical considerations about the use of gambling simulations in individuals with disordered gambling, and evidence that gambling severity is dimensional (Toce-Gerstein et al., 2003). However, within the context of substance addictions (namely alcohol dependence), the response to naltrexone is known to vary as a function of genetics (the OPRM1 polymorphism) (Ray and Hutchison, 2007) and family history of alcoholism (Krishnan-Sarin et al., 2007). Family history of alcoholism is also a predictor of a positive treatment response to naltrexone in pathological gamblers (Grant et al., 2008). In the study by Krishnan-Sarin et al. (2007), while naltrexone acted to decrease drinks consumed in a laboratory test in heavy drinkers with a family history of alcoholism, naltrexone actually *increased* drinking in those who were family history negative, similar to the effects observed here. The authors speculated that this effect may have been linked to individual difference in kappa-opioid action, which increase alcohol consumption in a rodent model (Mitchell et al., 2005).

In the most comparable study to the present experiment, Petrovic et al. (2008) found reduced brain responses to winning outcomes following opioid blockade in healthy participants, coupled with greater activation to monetary losses. However, the Petrovic et al. (2008) study (and also Drewnowski et al., 1995) used naloxone rather than naltrexone, and delivered intravenously rather than orally. The more rapid changes in brain concentrations associated with intravenous injection as opposed to oral dosing may cause divergent effects on behavior, as in the case of methylphenidate (Volkow and Swanson, 2003). Naltrexone may also exert partial agonism effects (Ignar et al., 2011), and along with naloxone and nalmefene, it is only moderately selective for the μ-opioid receptor, which may modify its effects on reward seeking behavior (Giuliano et al., 2012). As a third notable difference, the majority of past work in clinical groups has employed either subchronic (e.g. 7 day) dosing (e.g., Davidson et al., 1999; Drobes et al., 2004; Myrick et al., 2008) or slow-release depot formulations (Langleben et al., 2012a). Compensatory effects can occur in single-dose designs; for example, single dose citalopram treatment in healthy volunteers induced an impairment in reversal learning that was comparable to (rather than opposite to) effects observed in patients with major depression (Chamberlain et al., 2006). Nevertheless, the single administration of naltrexone used in the present study was seen to increase plasma prolactin levels, replicating Shaw and Al'Absi (2010). Given that prolactin release is inhibited by hypothalamic dopamine transmission (Freeman et al., 2000), a prolactin rise is presumed to reflect downstream dopamine blockade, indicative of overall opioid down-regulation.

In terms of the other gambling distortions under study, our findings were mixed. Consistent with the increased responsivity to wins, there was also an indication of enhanced confidence after winning streaks (i.e., increased “hot hand” effect). However, the motivational effects of near-miss outcomes were blunted in the naltrexone group. Given that the naltrexone effect on near-misses was restricted to a subjective rating (“continue to play”) and did not generalize to the psychophysiological measures, this result should be treated with caution. Moreover, the naltrexone group did not differ from placebo on two cardinal gambling distortions, the Gambler's Fallacy (on the roulette task) and the illusion of control (the manipulation of personal choice on the slot machine task), despite the fact that these distortions were robustly elicited in the overall study group. Related to the possibility that pathological gamblers may show a distinctive response to naltrexone, it is also conceivable that pathological and recreational gamblers may differ in their responses to gambling effects like near-misses (Habib and Dixon, 2010) or illusory control (Orgaz et al., 2013).

### Effects of haloperidol on gambling behavior

Prior research has shown that the stimulation of dopamine transmission can induce (Voon et al., 2009) and exacerbate (Zack and Poulos, 2004) gambling tendencies, as well as specific distortions including the sense of agency (relevant to the illusion of control) (Moore et al., 2010) and the behavioral response to near-misses (Winstanley et al., 2011). There is some evidence that these effects are D2-receptor specific (Zack and Poulos, 2007; Weintraub et al., 2010; Winstanley et al., 2011). Based on the argument by Frank and O'Reilly (2006) that lower doses of dopamine D2 receptor antagonists act preferentially on presynaptic D2 auto-receptors to *increase* dopamine transmission (see also Zack and Poulos, 2007), we predicted that low dose haloperidol would enhance the reactivity to win and near-miss outcomes on the slot machine task, and increase the influence of personal choice. We found limited support for these predictions, and haloperidol showed few effects in this study. The only statistically significant difference from the placebo group was on the pleasantness ratings on the slot machine task, where the haloperidol group showed increased pleasantness ratings after wins and increased ratings of unpleasantness after non-win outcomes. This effect was not corroborated by any change in physiological reactivity under haloperidol. It should also be noted that collapsing across treatment groups, the pleasantness ratings varied significantly as a function of personal choice (i.e., an Outcome × Choice interaction), but no 3-way interaction was evident with treatment group. We infer that the haloperidol group may have been more extreme in their affective ratings, but that this may not constitute a genuine drug action.

Notably, the lack of any observed effect of haloperidol on prolactin levels raises the possibility that the 2 mg dosage may not have been functionally active. In the study by Frank and O'Reilly, 2 mg haloperidol significantly increased prolactin levels in a cross-over design. While we note that our post-dose plasma sample was obtained slightly earlier (at 2.5 h) than the expected peak (at 3 h in Darby et al., 1995; at 4 h in Frank and O'Reilly, 2006), we also observed no cardiovascular or mood effects, unlike past reports (Zack and Poulos, 2007; Pine et al., 2010). A number of other studies have employed low doses of haloperidol (1–3 mg) in 3-arm studies that have included a group treated with the dopamine precursor levodopa (Pessiglione et al., 2006; Pleger et al., 2009; Pine et al., 2010; Oei et al., 2012). These studies have generally succeeded in demonstrating linear effects (i.e., haloperidol < placebo < levodopa) on reinforcement-related parameters, although in several instances, the specific haloperidol vs. placebo contrast was either non-significant (Pine et al., 2010), or not reported (Pessiglione et al., 2006; Oei et al., 2012). Of course, based upon the argument of presynaptic upregulation, an intermediate dose may exist where the presynaptic and post-synaptic actions cancel each other out. It is also recognized that both phasic and tonic components of dopamine signaling are implicated in reward-driven behavior, and that a presynaptic manipulation may primarily affect phasic firing (Grace, 1991; Niv et al., 2007). Overall, we find limited evidence for functional effects of the 2 mg dose, and the absence of a significant prolactin response is particularly concerning; we recommend that future studies in healthy participants opt for higher doses ≥3 mg.

### Limitations and conclusion

This study was the first to assess the effects of an opioid antagonist, naltrexone, and a dopamine D2-receptor antagonist, haloperidol, on gambling tendencies. The indications of increased gambling proclivity following naltrexone (increased physiological reactivity to wins, increasing confidence ratings on winning streaks) are at odds with the reported clinical efficacy of naltrexone in pathological gambling, although the non-clinical study population and single dose administration design necessarily limit any direct comparison. As a strength of the study, the two tasks were successful at inducing the key cognitive distortions of interest in the overall study group. While the group comparisons involved no correction for the multiple dependent variables (hence risk of Type I error), we sought to corroborate effects on behavioral measures and subjective ratings with the acquisition of event-related psychophysiology, which successfully demonstrated significant EDA and HR reactivity to wins and near-misses. We opted to use a 3-arm, parallel-groups design, because our tasks were not known to be suitable for repeated testing, although this decision had several consequences. First, the direct comparisons involved non-independent tests against the same placebo group, and some of the specific gambling effects (HR deceleration to wins, the hot hand effect) were not selectively evident in the placebo group. In addition, between-groups analysis limits any examination of individual differences in drug responses; for example whether dopamine or opioid effects varied with age or trait impulsivity (Zack and Poulos, 2009). As further limitations, we acknowledge that laboratory-based gambling simulations entail some compromises to ecological validity (Gainsbury and Blaszczynski, 2011). While our slot machine task delivered real monetary wins, which is important for establishing physiological arousal (Ladouceur et al., 2003), our tasks did not involve a variable wager. With regard to the limited effects of haloperidol on the gambling tasks, we highlight the non-significant change in prolactin as an indication that our low dose may not have achieved functional effectiveness, and as such, the null effects for haloperidol on the gambling tasks may say little about the relevance of dopamine signaling pathways to the neurobiology of gambling or the treatment potential of dopaminergic medications. However, the observed actions of naltrexone substantiate the relevance of opioid transmission to human decision-making and reinforcement processing, with treatment implications for a range of addictive and impulse control- related disorders.

### Conflict of interest statement

Luke Clark is a consultant for Cambridge Cognition Ltd. Ulrich Müller has been a consultant for Janssen Cilag and Eli-Lilly; he has received travel expenses and honoraria from the British Association for Psychopharmacology (BAP), Janssen Cilag, Eli-Lilly, Shire, UCB Pharma and the UK Adult ADHD Network (UKAAN) for talks at scientific and educational meetings. The other co-authors have no financial disclosures.
